# Interspecies Communication in Holobionts by Non-Coding RNA Exchange

**DOI:** 10.3390/ijms21072333

**Published:** 2020-03-27

**Authors:** Ana Lúcia Leitão, Marina C. Costa, André F. Gabriel, Francisco J. Enguita

**Affiliations:** 1 Faculdade de Ciências e Tecnologia, Universidade Nova de Lisboa, Campus da Caparica, 2829-516 Caparica, Portugal; aldl@fct.unl.pt; 2 MEtRICs, Faculdade de Ciências e Tecnologia, Universidade Nova de Lisboa, Campus da Caparica, 2829-516 Caparica, Portugal; 3 Instituto de Medicina Molecular João Lobo Antunes, Faculdade de Medicina, Universidade de Lisboa, Av. Prof. Egas Moniz, 1649-028 Lisboa, Portugal; marinacosta@medicina.ulisboa.pt (M.C.C.); andre.gabriel@medicina.ulisboa.pt (A.F.G.)

**Keywords:** non-coding RNA, inter-kingdom communication, microbiome, holobiont, hologenome, extracellular ncRNAs, miRNAs, metagenome, metatranscriptome

## Abstract

Complex organisms are associations of different cells that coexist and collaborate creating a living consortium, the holobiont. The relationships between the holobiont members are essential for proper homeostasis of the organisms, and they are founded on the establishment of complex inter-connections between all the cells. Non-coding RNAs are regulatory molecules that can also act as communication signals between cells, being involved in either homeostasis or dysbiosis of the holobionts. Eukaryotic and prokaryotic cells can transmit signals via non-coding RNAs while using specific extracellular conveyors that travel to the target cell and can be translated into a regulatory response by dedicated molecular machinery. Within holobionts, non-coding RNA regulatory signaling is involved in symbiotic and pathogenic relationships among the cells. This review analyzes current knowledge regarding the role of non-coding RNAs in cell-to-cell communication, with a special focus on the signaling between cells in multi-organism consortia.

## 1. The Concept of Cognitive-Based Evolution Beyond Post-Darwinism Theories

In the last 100 years, the postulates that were published by Darwin in his “Origin of the species” have been complemented by the increasing knowledge of Genetics and Molecular Biology. Darwin was responsible for the concepts of natural selection, but, most importantly, for the observation of the phenomenon of organism variation. Post-Darwinism theories of evolution relied on the incorporation of the concepts from Genetics into the theory of natural selection and variation, giving central importance to the genes: genes are responsible of evolution by random mutations using natural selection as a main driving force [1]. However, the increasing amount of data that have originated from static and dynamic genome analysis has modified the centrality of the gene in the evolutionary landscape.

Cognition-based evolution is a modern theoretical alternative for explaining the evolution of species in a landscape where genes are only cellular tools that are devoted to analyze and process environmental information [2]. Cognitive evolution is based on the assessment that any biological action is preceded by the existence of a source of information, and that cells must analyze this information and deploy the proper actions to maintain homeostasis [3]. All of the cells showed awareness, self-reference, and preferences at their own environmental limits and scale, sustaining critical homeostasis for life and being able to react, anticipate, socialize, and solve metabolic problems [3,4]. Following cognitive evolution, biology depends on the acquisition, distribution, and management of information by cells. Communication among cells and the establishment of cell-to-cell connections constitutes an essential way of acquiring better information, evaluating it, and responding to the often-ambiguous sources of biological and environmental data [5]. Evolution is not only driven by a random set of mutations controlled by natural selection, but also by an entangled network of interactions between the cells and the environment modulated by a complex information system that includes the individual cells and the surrounding biotic counterparts. Within this context, genetic information is only an information tool that can be used by cells to deploy and transmit information with the goal of maintaining cellular self-identity and homeostasis.

## 2. Holobionts in Cognitive Evolution: The Hologenome

Multicellular organisms are not singular entities, but rather a complex association of different organisms with diverse degrees of complexity, the holobiont [6]. Plants and animals are associated with microorganisms, and the nature of these associations affect the fitness of the holobiont within its living environment. Following cognition-based evolution, Rosenberg enunciated the “hologenome” theory based on the idea that the holobiont constitutes a unitary element for selection and evolution [7]. The hologenome can be consequently defined as the sum of all the genetic information resulting from the holobiont, which includes the hosting organism plus all the associated microbiota [7].

The hologenome concept can be considered to be a holistic view of genetics, where the animals and plants are multigenomic organisms. Genetic variations in the hologenome can cause variations in the hologenome phenotype that is driven by natural selection. Natural selection can work to remove deleterious mutations in the host or associated microbes while spreading advantageous genetic variations; in the absence of selection, the neutral spread of hologenomic variation through populations is an entirely stochastic process [8]. In a hologenome, there is considerable variation across many individual genomes that span the nucleus and organelles from the host and microbiome. Base pair mutation, horizontal gene transfer between holobiont components, recombination, gene loss and duplication, and microbial loss and amplification are all sources of hologenome variation. Because microbes are organisms characterized by their fast growth rates and mutation frequencies in their genomes, they must harbor an important part of genetic plasticity of holobionts. Although the hologenome concept redefines the structure and components of a complex living organism, they are compatible with the fundamental principles of Darwin’s and Wallace’s theory of evolution [8,9].

The currently limited understanding of hologenomes is based on the knowledge of binary relationships between organisms, namely host-pathogen interactions, but it needs to be expanded in order to reveal the true complexity of living systems [10]. Host-microbe interaction within the hologenome context is a key factor for evolution, supported by an entangled network of communications between holobiont components that are founded on metabolic and genetic signals [11]. Holobiont fitness and equilibrium needs the presence of transversal communication languages that are understood by all of its components [12]. Supported by the centrality of RNA molecules in all living organisms, the importance of an ancient RNA-based communication language needs to be considered within the holobiont scenario.

## 3. The Non-Coding RNA Language for Cell-to-Cell Communications

### 3.1. Diversity of RNA Species in Transcriptomic Output

In 1968, Francis Crick published a paper describing the role of the dynamics of genetic information in the evolution and the fundaments of the origins of the genetic code in living cells. This paper is considered to be the embryonic core of the “RNA world” theory [13]. The RNA world stands on the idea that RNA was the first “living molecule”, being in the origin of the evolution of all biological macromolecules and, consequently, acting as a biological platform from which other more complex biomolecules will arise [14]. RNA was favored in pre-biotic molecular evolution due to its intrinsic versatility and adaptability, and because it can carry sequence and structure information. In consequence RNA must be placed at the root of the molecular tree of life [15]. In most cases proteins have become more efficient to perform the same catalytic tasks, and they have been delegated to those functions along evolution, despite the existence of catalytic RNA molecules, as represented by ribozymes and the rRNAs. This fact denotes a clear Darwinian pattern of molecular evolution events, where a function is acquired and further improved and radiated into new ones by environmental pressure [15].

The dynamics of genomic output either in eukaryotic or in prokaryotic cells is largely determined by the pervasive transcription phenomenon [16,17]. Genome-wide transcription in all living cells is responsible for the biosynthesis of two main classes of RNA molecules: The coding RNAs will be translated to produce proteins, and the non-coding RNAs (ncRNAs) constituted by transcripts with reduced coding potential will not be translated [18]. ncRNAs are regulatory molecules that are generated either from specific transcriptional units or by the processing of already existing RNAs with alternative functions. In complex eukaryotic organisms, ncRNAs comprise most of the transcriptome, which suggests the importance of this second evolutionary tier of genetic output that might enable the integration and coordination of sophisticated suites of gene expression required for differentiation and development [19].

The non-coding transcriptome of eukaryotic cells includes several families of molecular species that are typically classified by their size, biogenesis pathways, and regulatory functions. The most widely used ncRNA classification criteria divides them in two families: short ncRNAs, comprised by molecules less than 200 nt in length, and long ncRNAs, a very diverse group of high-molecular weight ncRNAs, with more than 200 nt [20]. The short ncRNAs include the well-known groups of ncRNA involved in protein translation (the 5.8S and 5S rRNAs, and tRNAs) and RNA splicing and modification (snoRNAs), together with other regulatory ncRNAs, such as microRNAs (miRNAs), PIWI-interacting RNAs (piRNAs), and small interfering RNAs (siRNAs) [21]. miRNAs are one of the most versatile families of small eukaryotic ncRNAs composed by single-stranded short RNA molecules (19–23 nt) generated from the transcription and further processing of selected transcriptional units that act as negative regulators of the genomic output at the post-transcriptional level [22]. In eukaryotic cells, the family of long ncRNAs can be also divided in two subgroups: long non-coding RNAs (lncRNAs), transcriptional products of genomic loci with very limited coding potential that can regulate the genomic output at many levels from transcription to translation [23], and circular RNAs (circRNAs), covalently closed ncRNAs that result from non-canonical back splicing events of coding or non-coding transcriptional units that typically act as molecular sponges interacting with proteins or other ncRNAs [24]. The abundance and expansion of the repertoire of ncRNAs in higher eukaryotic organisms characterize them as a molecular players of evolution, because the organisms could not be dependent on a limited number of proteins or protein isoforms, but on a much larger set of genomic instructions that are embedded in trans-acting RNAs [25]. It is also very likely that dynamic ncRNA-regulatory networks can contribute much of the phenotypic variation that is observed between individuals and species and it can be involved in the response to internal and external stimuli [23].

In prokaryotic organisms, genome size and structure limit the heterogeneity of the transcriptional output when compared with eukaryotic cells. However, several ncRNA families have been characterized as relevant players in bacterial physiology and homeostasis. For instance, small bacterial ncRNA (sRNAs) are typically 50 to 400 nucleotides in length and, despite their lower abundance and diversity when compared with eukaryotic cells, they are important regulators of gene expression in specific conditions, like stress response, quorum sensing, and virulence [26,27]. Coleman and coworkers described the first evidence of a functional sRNA of bacterial origin with the characterization of micF, a non-coding RNA complementary to the Shine-Dalgarno sequence of the *E. coli* lpp mRNA that was able to silence its expression [28]. Bacterial sRNAs belong to two functional families: the cisRNAs originated by antisense transcription of coding genes that act over the parental coding frames by complementary base pairing and they are generally involved in processes, such as the maintenance of the copy number in plasmids or the regulation of operons [29] and the trans-encoded sRNAs that are generated by specific transcriptional units and involved in the post-transcriptional regulation of mRNAs [30]. Moreover, the recent developments of next-generation sequencing approaches for transcriptomic analysis allowed for the characterization of new populations of bacterial sRNAs derived from the post-transcriptional processing of other RNAs [26]. Because of their smaller size (16–25 nt), this family of bacterial sRNAs is often referred as “miRNA-like” and they have been characterized as co-adjuvant virulence factors in some pathogenic bacteria [31].

The importance of RNA molecules makes RNA an ideal player for the interchange of information between cells. There is a common ancient language encoded in RNA molecules, independent of their origins, which can be understood by cells and translated into metabolic actions. The putative roles of an RNA-based language are particularly interesting when the relationships between different species are analyzed. The role of RNA molecules, more specifically ncRNAs, in the interchange of information between cells is starting to be unveiled with the characterization of RNA-based communication mechanisms in binary systems, but more knowledge is required in order to understand the detailed functions of ncRNA regulatory networks in the context of holobionts [32,33].

### 3.2. Conveyors for ncRNA Transport between Cells

Valadi and coworkers observed RNA transfer between cells under physiological conditions and without involving the destruction of the cell structure for the first time in mouse and human cell lines in 2007 [34]. This functional transfer was mediated by extracellular vesicles, namely exosomes, containing diverse coding and non-coding RNAs. Exosome-mediated RNA transfer from mouse into human cells was sufficient to release coding RNA molecules that were translated to human proteins in the mouse cell line, but the exosomes were also loaded with numerous miRNAs that could have functional effects in the recipient cell [34]. Functional ncRNA transfer between tissues at the organism level was further demonstrated in animal models, showing the specific release of exosome-encapsulated miRNAs from niches, such as the adipose tissue, their transport across the animal body by circulating fluids, and their final regulatory action over distant cells [35].

Extracellular vesicles (EVs), which are the major conveyors of ncRNAs in their cell-to-cell journey, can be classified according to their diameter and their biogenesis pathways [36]. EVs that are generated by the exocytic pathway in eukaryotic cells are designated as exosomes and are 30–150 nm in diameter. Ectosomes or shedding microvesicles are generated by the outward budding of cell membranes and they are typically larger than exosomes with a diameter between 100–1000 nm. Later stages of apoptosis are responsible for the generation of a third family of EVs, the apoptotic bodies, which are a very heterogeneous family of vesicles that present diameters that ranged from 50–5000 nm [37]. Bacteria are also able to generate nano-sized EVs either constitutively or as a response to environmental stimuli [38]. EVs of bacterial origin were initially described in Gram negative microorganisms as the result of the shedding of the outer-membrane and they were consequently designated as outer membrane vesicles (OMV). Recent evidence also showed that Gram positive bacteria can produce EVs, but the mechanism for the biogenesis and release of those microvesicles are still not completely characterized [26,39].

**Figure 1:**
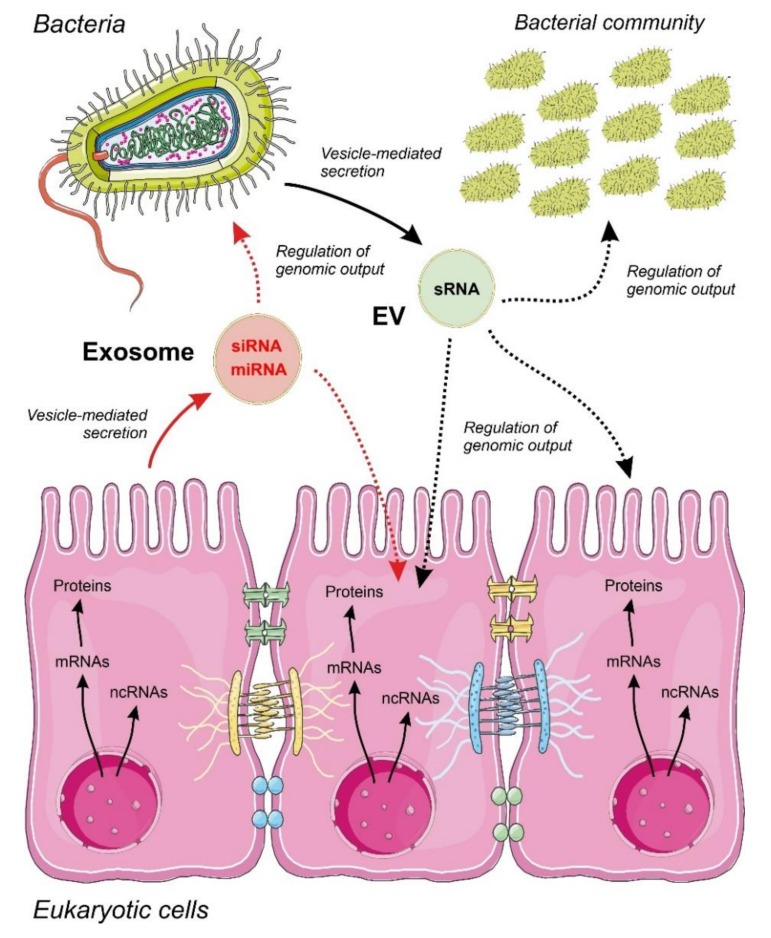
Proposed model for inter-regulatory networks mediated by non-coding RNAs (ncRNAs) secreted within membrane-containing vesicles in a binary system composed by a eukaryotic epithelium and an associated bacterial community. Small regulatory ncRNAs produced by eukaryotic cells (miRNAs and siRNAs) and bacteria (sRNAs) are selected, sorted and secreted by vesicles acting as regulators of the genomic output crossing cell boundaries. The specificity and detailed mechanisms involved in ncRNA selection and sorting by the cells are largely unknown. The widespread regulatory language transported and exerted by ncRNA molecules allow a cross-kingdom communication and an efficient interaction between different cell types. Metabolic flow from genomic information is depicted by continuous arrows and regulatory events controlled by ncRNAs by dotted arrows.

Independent of their biological origin, there are still many open issues regarding the functions of EVs as carriers of ncRNAs, namely those that were related with the mechanisms used by the cells to select and process ncRNAs as vesicle cargo (Figure 1). The current accumulated knowledge about the mechanisms of ncRNAs sorting into secretory vesicles is focused on miRNAs in human and mammalian cells. Experimental evidence showed that the miRNA sorting into exosomes and other vesicles is mediated by RNA-binding and cytoskeleton proteins, following patterns that appear to be cell specific [40,41,42]. Interestingly, in human cells, the external perturbation of the expression of individual miRNAs or their cognate targets promotes bidirectional miRNA relocation from the cytoplasm to multivesicular bodies, controlling the miRNA sorting to exosomes [43]. The mechanisms of sorting ncRNA into vesicles remain unknown in lower eukaryotes and bacteria.

### 3.3. Molecular Machinery for Understanding the External ncRNA Message

Once the extracellular signals that were transported by ncRNA reach the target cell, they need to be translated into functional actions to generate a physiological response. Within the holobiont context, the ncRNA-mediated signals need to be efficiently understood by the target cell and ideally by different target cells. The efficient communication by ncRNAs is ensured by the plasticity of RNA molecules, their presence in all living cells, and shared mechanisms of action among distinct organisms. The small RNAs produced by eukaryotic (miRNAs and siRNAs) [44,45] and prokaryotic cells (sRNAs) illustrate the inter-cell communication that is mediated by ncRNAs [46,47]. The regulatory roles of other ncRNA families in cross-species communication, such as circRNAs or lncRNAs, remain unknown.

In eukaryotic cells, external small RNAs that are transported by EVs are internalized either by vesicle endocytosis or by membrane fusion and posterior release of vesicle cargo into the cytoplasm. The mechanisms of a hypothetical targeting of ncRNA-containing vesicles to specific cells remain unknown. Signals that are transported by small ncRNAs (miRNAs, siRNAs or bacterial sRNAs) and targeting eukaryotic cells are expected to function by the insertion of these external molecules into the canonical regulatory pathways that are mediated by ncRNAs in the cell. Experimental evidence suggests that foreign regulatory small ncRNAs would function by base pairing to their molecular targets, which are usually mRNAs as well as ncRNAs [48]. It is important to note that the regulatory action of any small ncRNA is strictly dependent on the presence of the corresponding cognate target in the recipient cell. The same small ncRNA could have different regulatory effects, depending not only on the targeted cell, but also on its physiological state. Moreover, small ncRNAs would also need the help of specific RNA binding proteins that will be responsible for the final regulatory action.

In eukaryotes, small ncRNAs are recognized by conserved RNA-binding proteins that belong to the argonaute family (Ago) [49]. Canonical and Ago-like proteins are encoded by the vast majority of eukaryotic genomes from fungi [50,51] to plants [52,53] and animals [54], ensuring a molecular background for the action of internal or external small ncRNAs [55,56]. In the cytoplasm of animal cells, a member of the argonaute protein family Ago2 is a key component of the RNA-induced silencing complex (RISC), which the base complementarity between the small RNA and its target will guide. If the complementarity between the small ncRNA and the target is close to 100%, then the endonuclease activity of Ago2 will be responsible for the targeted cleavage of the target transcript that will be further degraded by RNases. In the case of partial complementarity, the canonical miRNA pathway will induce a RISC-mediated translational repression by a mechanism that often involves transcript deadenylation and further decay [22,57]. In plants, Ago1 and Ago2 proteins are generally involved in sRNA-mediated gene silencing, which occurs by target RNA degradation due to the high degree of complementarity between the RNA molecules [58]. Solid experimental evidence supported the function of externally-originated small RNAs in order to regulate gene expression within different eukaryotic hosts [59,60].

Bacteria also have regulatory networks that are centered on small ncRNA molecules. The first evidence for the characterization of the protein players of these RNA regulatory networks pointed to the importance of the Hfq protein, a small RNA chaperone that was detected in association with small ncRNAs and mRNA transcripts [61,62]. Further experimental data described the additional existence of ribonucleoprotein complexes that were integrated by the Hfq chaperone together with RNase E that were responsible for the post-transcriptional silencing and degradation of target mRNAs by the action of specific families of complementary sRNAs [63,64]. Hfq-dependent RNA regulatory networks have been involved in virulence and pathogenesis processes in several bacteria [65,66], but they are also expected to be involved in the regulatory action of exogenously acquired ncRNAs [44,67]. In contrast to eukaryotic cells, the effect of bacterial ncRNAs over mRNA transcripts is different from canonical miRNA/siRNA regulation and it could also include an enhancement in the stability and expression of the targeted transcripts [44,67].

## 4. Cross-Kingdom Communication Mediated by ncRNAs in the Holobiont Context

The characterization of multi-species consortia is a difficult technical enterprise due to their intrinsic dynamic nature exemplified not only by the different cross-organisms relationships, but also by the direct interaction with environmental stimuli. As a first approach, the study of the distribution of the different holobiont species could be a valuable strategy for characterizing the population of the consortium. Initial attempts were based on the sequencing of 16S rRNA to characterize the presence of different species within multi-species consortia [68], but the development of next-generation sequencing techniques rapidly changed the paradigm. Metagenomics objectives rely on the use of massive sequencing technologies in order to characterize the genomic pool from natural sources [69] and allow for the deep characterization of hologenomes [69]. However, the inherent nature of the DNA limits the information regarding the physiological status of a multi-species consortium that is extracted from genomic data. Metatranscriptomics studies allow for the characterization of multi-species within an holobiont context by taking advantage of the dynamics of transcription [69]. Relevant examples of the use of metatranscriptomic approaches have been applied to the characterization of holobiont dynamics in response to different environmental stimuli in humans [70], insects [71], and corals [72].

However, the metatranscriptomic studies are still only focused on the individual population dynamics that are based on the study of the protein coding transcriptome. Further studies need to fulfill the lack of genome-wide information about the dynamics of the non-coding transcriptome and the vertical and horizontal transfer of ncRNAs between holobiont members [73]. The current knowledge about the roles of ncRNAs in communication between holobiont species is mainly based on pathogenic relationships and it has been characterized using model systems, but this knowledge is still valuable for understanding the subjacent mechanisms of the holobiont homeostasis. In the following sub-sections, we will describe several examples of ncRNA-mediated crosstalk between species within holobionts, focusing the targeted cell type.

### 4.1. Communication by ncRNAs Acting within Eukaryotic Cells in Holobionts

In a multi-species association, the most complex organism is considered to be the host of the holobiont, acting as a platform to which all of the saprophytic, symbiotic, or pathogenic organisms are associated. The host organisms are typically higher eukaryotes, ranging from plant to animals, which are dependent on the establishment of productive relationships with other organisms [32]. Any perturbation in the host-associated organisms, usually recognized as the “microbiota”, will lead to an alteration of the holobiont phenotype by a network of entangled metabolic and functional interactions within the consortium members [74]. In the case that those interactions are mediated by ncRNAs, eukaryotic cells have complex machinery to decode the information carried by these molecules into a metabolic response that could eventually lead to a positive or negative association between the interacting species. The role of ncRNAs in holobiont fitness remains elusive, because the current data demonstrating the fluctuations in the holobiont transcriptional output are usually from observations of dysbiosis events [72,75,76] or host-pathogen interactions [77,78,79]. In the following sub-sections, we will analyze the current experimental data showing the central role of ncRNAs in the relationships among holobiont cells.

#### 4.1.1. Corals

**Figure 2:**
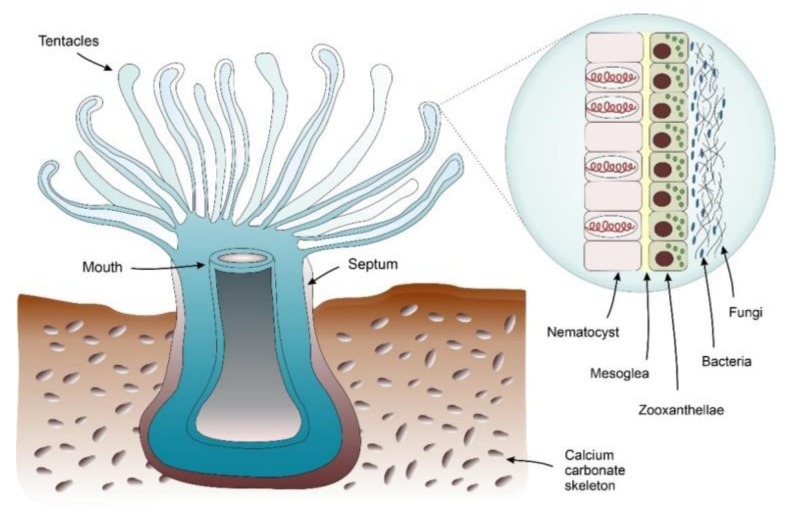
Corals are holobiont models where the intricate functional relationships between their members are very evident. As a primary association the nematocysts are associated with algae, which are responsible for the photosynthetic activity of the holobiont [75]. Moreover, recent evidence also showed the presence of an additional layer of biological complexity integrated by saprophytic fungi and bacteria that colonize the surface of the coral and also the Calcium carbonate skeleton [81]. Environmental events leading to an imbalance of the microbial populations will result in holobiont imbalance, phenotypically observed as a coral bleaching that it is often followed by an increase in fungal colonization [85].

Corals are considered to be complex biospheres that formed by the association of hundreds of different eukaryotic and prokaryotic species, where an entangled and complex network of interactions is established (Figure 2) [80]. Corals harbor a community of organisms that are composed by thousands of bacterial and fungal phylotypes in an association that could be geographical and temporally dependent, and able to react as a community in response to environmental changes [81]. The ability of the coral to adapt itself to environmental biotic or abiotic stress led to the “probiotic hypothesis” [82]. This hypothesis relies on the existence of a dynamic relationship between corals and symbiotic microorganisms in different environments that selects the most advantageous coral holobiont configuration, depending on the environmental conditions. Recent evidence suggested a co-evolution phenomenon between the coral host and its associated microbiota, but the nature of this symbiosis and its involvement in coral resilience and homeostasis remains unclear [83,84].

Understanding the complexity of the coral holobiont would need coordinated efforts to apply advanced molecular techniques in the characterization of different corals from diverse environments [81]. In this context, the Tara Pacific consortium intends to provide a baseline for coral “omics” data worldwide with the aim of characterizing novel biodiversity and unraveling the molecular links between genomes, transcriptomes, metabolomes, microbiota, and holobiont functions in coral reefs [86]. Population dynamics within the coral holobiont in response to biotic or abiotic stress has been the subject of deep investigation in the past years due to the ecological importance of corals [72]. Metatranscriptomic analysis of well-established coral populations showed a dynamic RNA expression pattern that can be corelated with the incidence of coral diseases, such as the growth anomaly [87] and the depletion of algae symbionts, also known as “bleaching” [76]. In both cases, the holobiont phenotype is characterized not only by a decreased biodiversity in its components, but also by a specific enrichment in some microorganisms. At the molecular level, the imbalance in the holobiont populations resulted in an increased expression of coding genes that are involved in immune and stress responses, as well as in genes related with the defense against free radicals [72,87,88].

The roles of ncRNAs in the coral holobiont fitness are not known, but preliminary data support the idea that they could act as modulators of the biotic or abiotic stress responses. One of the first evidence of the presence of functional ncRNAs in corals was obtained from small RNA-seq experiments that were performed in *Stylophora pistillata*. Liew and coworkers discovered at least 50 miRNAs from the coral, five of which (miR-100, miR-2022, miR-2023, miR-2030, and miR-2036) are also conserved in other metazoans [89]. In *Acropora digitifera*, RNA and small RNA-seq experiments were also used to characterize the presence of a non-conserved and thermally-responsive miRNA that putatively regulates genes that are involved in a general abiotic stress response, including protein-coding genes that were related with gene expression regulation, DNA repair mechanisms, tissue morphogenesis, and signaling [90]. Other families of ncRNAs, such as lncRNAs, have also been recently described as putative players in the observed coral responses due to microbiome imbalance. In *Protopalythoa variabilis* and *Palythoa caribaeorum* transcriptome-wide sequencing studies detected 11,206 and 13,240 expressed lncRNAs, respectively, some of them being conserved in higher eukaryotes [91]. Further investigation of differentially expressed lncRNAs in healthy colonies and individuals undergoing natural bleaching indicated that specific up-regulated lncRNAs in *P. caribaeorum* could act as post-transcriptional modulators of the Ras-mediated signal transduction pathway and components of the innate immune-system, as a part of the molecular response of coral bleaching [91].

miRNAs have been also associated with the establishment of endosymbiosis in corals, despite their involvement in stress responses. In the sea anemone *Aiptasia*, a model system for cnidarian-dinoflagellate endosymbiosis, transcriptomic, and small RNA-seq experiments showed that miRNAs are differentially expressed in response to endosymbiont infection. Moreover, Ago2 cross-linking and further immunoprecipitation identified miRNA binding sites on a transcriptome-wide scale and found that the targets of the miRNAs regulated in response to symbiosis include genes that were previously implicated in biological processes related to *Symbiodinium* algae colonization, suggesting the miRNA-mediated modulation of genes and pathways during the onset and maintenance of cnidarian-dinoflagellate endosymbiosis [92].

The isolated data regarding the role of ncRNAs controlling the responses of coral holobiont components against diverse stimuli implicate these ncRNAs in the control of the coral homeostasis. The transverse position of ncRNAs in biology and their demonstrated physiological roles within the coral components also suggested that ncRNAs could act as molecular signals within the holobiont, resembling the already known role of some secondary metabolites that are produced by coral pathogens to hijack its defenses and colonize tissues [85,93]. However, the horizontal transfer of ncRNAs between coral holobiont members has not been yet demonstrated [94].

#### 4.1.2. Plants

Plants are very interesting hosts from the point of view of the inter-species communication, because they harbor two well-differentiated niches; the root and the aerial structures that contact different environments [95]. In the beginning of the 20th century, Hiltner hypothesized about the existence of a protection mechanism that was based on the presence of specific microorganisms that would protect roots from an external infection [96]. These original hypotheses were recently confirmed by metagenomic analysis, showing the specific enrichment of microbial populations in the root environment, different from the observed in the plant leaves, and related to the plant holobiont fitness [97]. The species diversity of plant microbiota are higher in the exposed structures and lower in the roots [98].

External stimuli can trigger a plant phenotypic response that is observable in the dynamics of the host and associated microbial populations, but also in the genomic output of the individual holobiont components. For instance, metatranscriptomic studies of *Salix alba* roots that were exposed to petroleum hydrocarbons determined that the external abiotic stress was responsible for a population shift of the fungal communities that are associated with the tree roots. Under abiotic stress, root-associated fungi shifted from Ascomycota to Basidiomycota genera, and this biological shift was also accompanied by an increase of specific biofilm-forming bacteria responsible for the reduction of contamination stress [99]. Species-wide transcriptomic analysis was also employed in order to characterize the root-associated bacterial population dynamics in response to drought [100]. In both cases of abiotic stress, next-generation sequencing data showed a dynamic correlation between holobiont populations and hologenome output. The detailed characterization of the molecular mechanisms governing these interactions will require strategies that are based on the concepts of systems biology, as recently reviewed by Rodríguez and coworkers [101]. Among the different actors that are involved in plant-microbiota interactions, phytohormones are essential players in plant biology; they are able to control not only the homeostasis of the associated microbiota, but also regulate plant growth and differentiation. Phytohormones, such as salicylic acid, mediate the plant defenses to biotrophic pathogens, whereas jasmonic acid and ethylene are responsible for the plant responses against necrotrophs and insects [102]. Other hormones such as the auxins and gibberellins control plant growth [101]. Interestingly, phytohormones are also vehicles of bidirectional communication between the plant host and its microbiota, as exemplified by the strigolactones, hormones that are produced by the plant root during nitrogen starvation and attract symbiotic fungi to the rhizosphere [103,104]. Interestingly, some fungi have also acquired the capacity of producing phytohormones, such as gibberellins, which induce plant growth under abiotic stress conditions and facilitate root colonization [105]. Phytohormones are included under the family of the small organic compounds, but their functions as bidirectional communication signals could be resembled by extracellular ncRNAs, as was demonstrated by Wang and coworkers [48] in the analysis of the interaction between fungal pathogens and plants. During surface and tissue colonization, the fungal plant pathogen *Botrytis cinerea* produces siRNAs by the action of the Dicer-like proteins DCL1 and DCL2 over long double-stranded RNAs. These siRNAs are transported to the plant tissues silencing specific genes that are involved in the immune response against the pathogen. Additionally, tomato plants that are infected by *B. cinereal* are able to counteract the fungal siRNA action by the use of small ncRNAs that target and silence *DCL1* gene transcript in a bidirectional crosstalk mechanism that regulates pathogen colonization and progression [48].

**Figure 3:**
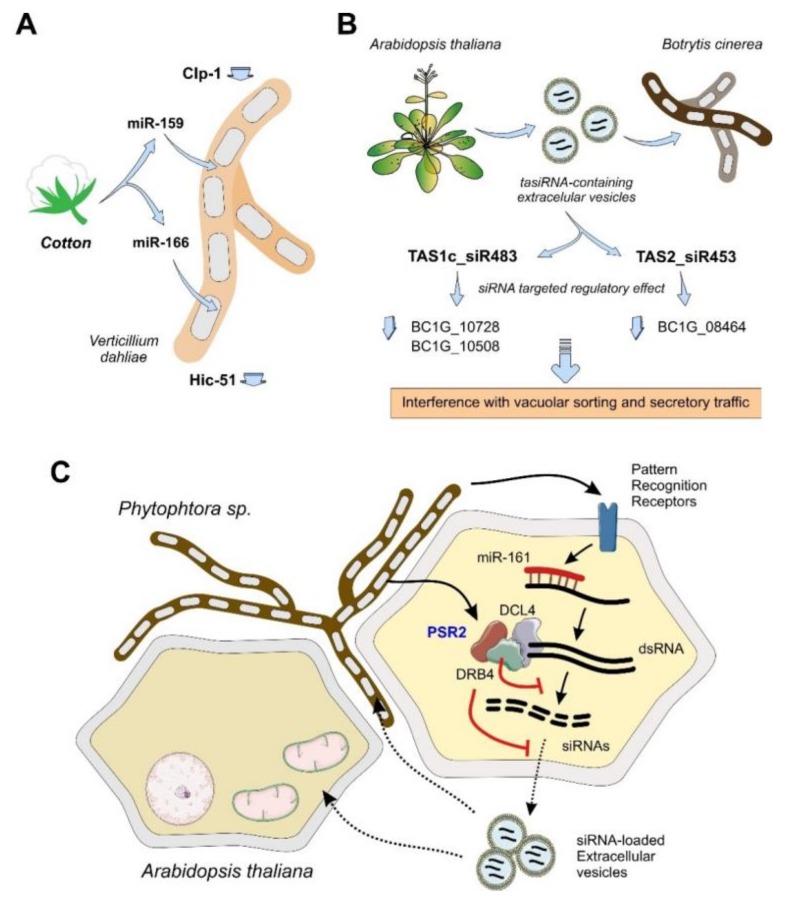
In plant holobionts, the intervention of pathogenic species could be responsible for holobiont imbalance [102]. In this context, ncRNAs are essential players for the relationships established between the host and the colonizing pathogens. (**A**), cotton plants counteract infections by the fungal pathogen *V. dahliae* by secreting specific miRNAs (miR-159 and miR-166) that inhibit the expression of the virulence factors Clp-1 and Hic-51 [108]. (**B**), *A. thaliana* plants are able to control the growth of pathogenic fungi as *B. cinerea* by using a defense mechanism that is based on tasiRNAs (a specific group of regulatory ncRNAs generated by the processing of long dsRNAs by Dicer-like enzymes), that will target a group of genes required for vesicle trafficking and secretion in the fungal pathogen. Vesicle trafficking is essential for fungal colonization of the plant, and the regulatory effect of the plant tasiRNAs will result in a control of the invading pathogen [111]. (**C**), fungal pathogens from the *Phytopthtora* genus have an extremely sophisticated ncRNA machinery for a functional crosstalk with their host plants. Plant cells can recognize the colonization by the fungal pathogen by the involvement of surface pattern recognition receptors (PRRs). The PRRs will lead to an overexpression of miR-161 that will be responsible for the biogenesis of siRNAs via dsRNA amplification and a Dicer-dependent mechanism. The siRNAs can target the genome of the infecting fungus when transported by extracellular vesicles. The *Phytophtora* fungus, can counteract this defense mechanism by the secretion of PSR2, a protein that inhibits the production of defensive siRNAs by interaction with the Dicer-associated dsRNA processing complex [112].

A specific gene expression pattern, including ncRNAs, characterize plant responses against biotic or abiotic stress [106]. Plant miRNAs constitute an important population of the ncRNA response against pathogens, and some of them are known to be involved in the regulation of the defense mechanism to prevent pathogenic infection. This is the case of the miR-169 family of miRNAs, whose intracellular levels have been correlated with the susceptibility to infection by fungi that is observed in some plants like *Musa acuminata* [107]. Moreover, plants are also able to secrete some of the infection-responsive miRNAs, while using them as a direct defense mechanism against fungal pathogens by a RNAi-like mechanism. For instance, cotton plants secrete miR-166 and miR-159 as a defense mechanism against the infection by *Verticillium dahliae*, targeting the mRNA transcripts of the fungal virulence factors Clp-1 and Hic-51 [108]. Inside the pathogenic fungus, the complementarity between the plant miRNAs and mRNA will induce a RNAi-like mechanism and a further targeted degradation of the coding transcripts, resulting in a decreased virulence of *V. dahliae* (Figure 3A). Similar results were described in the infection of wheat by *Fusarium graminearum*, where the plant miR-1023 targets and silence an alpha/beta hydrolase related with the virulence of the fungus [109]. Based on these results, Gabriel and coworkers postulated a general defense mechanism that is based on plant miRNAs against fungal pathogens that would target specific genes in the fungal transcriptome to silence them [110].

Interestingly, the plant hosts have unique strategies to increase the signals mediated by small ncRNAs. For instance, plants can amplify miRNA-targeted mRNAs by an RNA-dependent RNA polymerase to generate dsRNAs that will be further processed by a Dicer-like enzyme producing siRNAs that are responsible for gene silencing of the original mRNA transcript [113,114]. The generated dsRNAs are generally designated as trans-acting siRNAs or tasiRNAs, and their biogenesis depends on the presence of the plant Dicer-like enzyme DCL4 [115] and RNA-binding proteins, such as DRB4 [116]. Some plant-pathogen interactions are regulated and mediated by tasiRNAs. For instance, when the fungus *Botrytis cinerea* infects *Arabidopsis thaliana*, the host triggers a defense response that is based on vesicle-secreted tasiRNAs (Figure 3B). Two *A. thaliana* regulatory tasiRNAs, TAS1c_siR483 and TAS2_siR453 exert a regulatory function over the fungal transcriptome by silencing specific gene transcripts that are involved in vacuolar sorting and secretory vesicle trafficking, resulting in a decreased virulence of the infection [111].

Membrane proteins, called pattern recognition receptors (PRRs), modulate relationships between the plant host and the associated microbiota. These proteins can recognize surface features present in the holobiont microbiota and are linked to the biosynthesis of molecular signals to control the growth of potentially pathogenic organisms. The role of PRRs associated with the production of small ncRNAs is clearly illustrated with the bidirectional relationship that was established between the plant pathogen *Phytophtora* sp and *A. thaliana*. This relationship is mediated by miRNAs and tasiRNAs. During an infection by the fungus, PRRs will recognize the presence of the pathogen and induce the overexpression of miR-161. This miRNA is involved in the immune response against pathogens and it will target specific mRNA transcripts that will be amplified by the tasiRNA pathway (Figure 3C). The generated tasiRNAs will be loaded into extracellular vesicles and directed to the infecting fungus, silencing virulence genes. Interestingly, the *Phytophtora* sp is also able to counteract the tasiRNA-mediated plant defense mechanism by the production of PSR2 protein (*Phytophtora* Silencing Repressor protein 2) that will directly block the production of the defensive tasiRNAs in the plant by inhibition of the dsRNA processing complex [112]. Despite the characterization of this crosstalk mechanisms mediated by siRNAs in host-pathogen relationships, the role of PRRs and their involvement in the ncRNA-mediated signaling for the homeostasis of the whole plant holobiont remains unclear.

#### 4.1.3. Mammals

The presence of territories that are clearly related with the biodiversity in the associated holobiont components characterize the anatomic and physiological organization of mammals and other complex animals. Internal cavities, such as the intestine, contain the most diverse associated microbiota, ensured by the microorganisms present in food, but also by the relatively protected environment when compared with other biological niches, such as the skin [117]. The intestinal microbiome is essential for the mammalian holobiont fitness, because it provides relevant metabolic and immune advantages to the host [118,119]. Enzymatic activities of gut microbes contribute to the degradation of indigestible food components and they are vital to the production of certain essential nutrients, such as vitamin K [120]. The microbiome connection to the host immune system is extremely important, and this relationship is regulated to both enable immune tolerance of dietary and environmental antigens providing protection against potential pathogens [121,122]. The intestinal microbiome directly protects the host from exogenous pathogens by a competitive association to epithelial cells and indirectly, by triggering immune responses [123,124]. Selected human and animal studies clearly showed the existence of communication axes between the intestine and other organs that are extended beyond the gut, and that contribute to the function and dysfunction of distant organ systems [125,126]. Gut microbiome dysbiosis has been correlated with several types of tumors, suggesting the direct connection between the microbiota and tumor proliferation [127,128].

Pathogen-induced dysbiosis in humans and other mammals results in a coordinated response from the host with the objective of eliminating the pathogenic agent and restoring the system fitness, and they are characterized by a specific gene expression pattern from the host that includes ncRNAs [129]. The role of host specific families of ncRNAs, such as miRNAs, in reshaping the immune response against pathogens has been reviewed elsewhere [130]. Surface pattern recognition receptors (PRRs), such as the Toll-like receptors (TLRs), can recognize structural features of bacterial and fungal pathogens, which include single- and double-stranded RNAs and can trigger a cascade of signals that often involves the production of cytokines [131]. Some bacterial pathogens can hijack the defensive system by inducing the expression of specific miRNAs that can extend their survival or downregulate the production of cytokines. This is the case of *Mycobacterium tuberculosis*, which can induce the expression of miR-32-5p and miR-124, both negative regulators of pro-inflammatory cytokine production in macrophages [132]. Another ubiquitous host miRNA, miR-146a, is induced by several pathogenic bacteria, such as *Escherichia coli*, *M. tuberculosis*, and *Helicobacter pylori*, causing a negative regulatory effect in the LPS-mediated response, cytokine production, endocytosis, and vesicle trafficking [133].

Some intracellular bacterial pathogens can also deploy molecular attacks on the host cell by using ncRNAs that are generated from specific transcriptional units, demonstrating the role of RNA molecules as ubiquitous regulators [134]. For instance, *Salmonella typhimurium* synthesizes a ncRNA designated as Sal-1, which is processed inside the host infected cell by a mechanism involving Ago2 and that resembles the final steps of miRNA biogenesis in eukaryotic cells [135]. The processed miRNA-like bacterial ncRNAs will target the transcript from the iNOS gene, an inducible Nitric-oxide synthase, thereby attenuating the host cell iNOS/NO-mediated anti-microbial capacity and increasing the bacterial proliferation [136]. Similar examples of bacterial ncRNAs transferred to eukaryotic cells can be found in lung infections by *Pseudomonas aeruginosa* [47] and the periodontal and mouth infections that are caused by *Treponema denticola*, *Aggregatibacter actinomycetemcomitans*, and *Porphyromonas gingivalis* [31]; the colonization of the infectious bacteria is made on the mucosal surface of the lungs or the mouth and the ncRNAs are delivered to the host cell via OMVs.

Parasitic infections are other examples of host-pathogen interactions that involve the transference of ncRNAs to a mammalian host [94]. For instance, in the blood stage of malaria, the infected erythrocytes are the source of EVs whose cargo is composed by a mixture of molecules, including host and parasite ncRNAs (rRNAs, snoRNAs, and fragments of tRNAs) [137]. The *Plasmodium*-derived EVs that originated from erythrocytes are transferred to endothelial cells, suggesting a possible communication mechanism mediated by signaling molecules and ncRNAs [137,138]. Very recently, Dandewad and coworkers described the retrograde mechanism that involves a transport of human miRNA-RISC complexes into *Plasmodium falciparum* cells and a further regulatory mechanism of the parasite mRNA targets [139]. These combined mechanisms demonstrate an intimate molecular cross-communication between the malaria parasite and its host where ncRNAs are important players and could be extensive to other protozoa [140,141] and helmintic parasites [142].

### 4.2. Communication by ncRNAs Acting within Bacteria in Multi-Species Consortia

Regulatory effects that are exerted by ncRNAs are not exclusively observed in eukaryotic cells, but also in bacteria [143]. During the last decade, new data showed the existence of a very complex panoply of ncRNAs in bacteria, including medium RNA size species (40 to 200 nt), which could act in cis or trans and also small ncRNAs that resemble the mechanism of action of eukaryotic miRNAs [27]. Consequently, bacteria are able to decode ncRNAs from either internal or external sources [44]. In complex holobionts, like mammals, the disruption of the symbiosis of microorganisms with the host results in disbiosys, defined as detrimental imbalance of microbiota related with a pathological phenotype [144]. In the gastrointestinal tract of mammals, the gut microbiota composition is directly related with the expression and secretion of miRNAs from the epithelial cells to the intestinal lumen. The results obtained using germ-free model animals suggest that fecal miRNAs are likely to act as mediators in the communication between the microbiota and the host in several conditions, such as colorectal cancer or inflammatory Bowel disease, and also in the regulation of the homeostasis of the holobiont [45]. Interestingly, functional relationships between the gut microbiota and the mammalian host can be further extended, including a functional axis that connects the gut to the brain and the immune system; however, the role of ncRNAs as communicators along the gut-brain axis has not been resolved [125,126].

**Figure 4:**
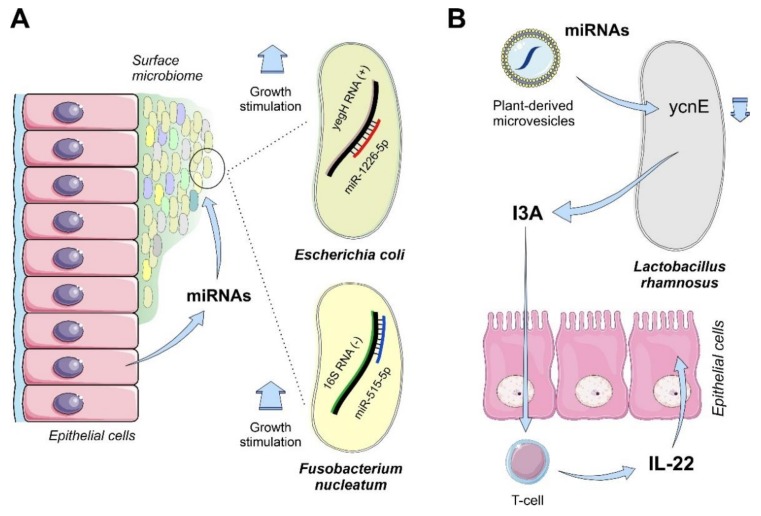
Selected models of the functional effects of exogenous ncRNAs on bacterial cells in the human intestinal microbiome. (**A**), epithelial cells can interact with the mucosal microbiome by the secretion of miRNAs that are enriched in the intestinal lumen. Among these miRNAs, miR-515-5p and miR-1226-5p were characterized as direct regulators of the 16S RNA and yegH RNA from *F. nucleatum* and *E. coli*, respectively. In vitro experiments showed a direct correlation between miRNA-target interaction and the increase of growth of these members of the microbiota consortium [44]. (**B**), plant-derived extracellular vesicles containing miRNAs from food intake are responsible for the induction of an interleukin-mediated response that keeps the homeostasis of intestinal cells and counteract bacterial colitis. The regulatory effect observed in *L. rhamnosus* involves the direct regulation of the mRNA transcript of ycnE which results in an increased production of the tryptophan metabolite indole-3-carboxaldehyde (I3A) by the bacteria. The I3A metabolite induces the production of interleukin 22 (IL-22) by lymphocytes that contributes to the improvement of the intestinal barrier function [147].

Bacteria from gut microbiota can capture small ncRNAs produced and secreted by the intestinal cells despite the lack of knowledge about the mechanisms that are involved in the internalization of external ncRNAs. In humans, miR-515-5p is a tumor suppressor controlling cell proliferation in breast cancer cells by regulating the sphingosine kinase SK1 transcript [145] and cell migration by targeting the MARK4 kinase mRNA [146]. This tumor suppressor miRNA can also act as an extracellular messenger when it is secreted by intestinal cells, by selectively targeting specific bacterial gene transcripts to stimulate the fitness of the microbiota. Liu and coworkers demonstrated the direct stimulation of proliferation by miR-515-5p together with miR-1225-5p in *E. coli* and *Fusobacterium* in the intestine. These miRNAs target the *yeg*H and 16S RNA gene transcripts (Figure 4A) [44]. The underlying mechanisms, including the molecular players of this stimulation of bacterial proliferation, are unknown.

Interestingly, plant-derived miRNAs that are ingested by mammals and transiting through the intestinal track within microvesicles can be also connected with the homeostasis of the intestinal mucosa. Animal models connect the dietary plant miRNAs to the production of interleukin IL-22 by T-cells that are associated with the intestine basal membrane and with an improvement of the fitness of the intestinal mucosa. miRNAs that are transported by plant microvesicles within the intestine can target the *ycn*E gene transcript in *Lactobacillus rhamnosus*, inducing the accumulation and secretion of indole-3-carboxyaldehide (I3A) by the bacteria. I3A will be responsible for the secretion of IL-22 by T-cells and a further increase in the epithelial membrane integrity, protecting the intestine from the colonization of pathogenic microorganisms (Figure 4B) [147].

## 5. Conclusions and Future Perspectives

The RNA world hypothesis describes the centrality of RNA molecules in evolution, as they constitute a common functional language that could be understood by all living organisms. Despite the evolutionary constraints that favored proteins for some specific functions, where they are more efficient, RNA molecules are very flexible, since they harbor sequence and structure information. The intrinsic plasticity of RNA is demonstrated by the ubiquitous presence of ncRNAs as transcriptional products of virtually all the living genomes. The ability of ncRNAs to act as functional messengers that cross the boundaries of different species is perfectly connected with the idea of the existence of hologenomes as basic units of information in evolution. Within this context, mobile ncRNAs may constitute an important and flexible mechanism of genetic information interchange between different species and, in consequence, they will be involved in the genetic plasticity of hologenomes.

The dynamics of hologenomes have been the subject of intense study in the last five years and they have been powered by the development of new hardware for high-throughput transcriptomics and computer algorithms for data analysis. Metatranscriptomic analysis of holobionts that were exposed to different environmental conditions were used for the molecular characterization of the population dynamics within the multi-species consortia. However, the characterization of the molecular signals that are involved in the communication between the host and the associated holobiont microbiota still constitutes an unmet need. The role of mobile ncRNAs in the communication between holobiont members has started to be unveiled by the study of dichotomic relationships, namely those that involve host-pathogen or host-symbiont relationships. In host-pathogen relationships, mobile ncRNAs that are transported by membrane-containing conveyors have been characterized as playing important roles either as a pathogenic signal or as defense mechanisms; however, their roles in the homeostasis and holobiont fitness are still not clear.

The complete characterization of ncRNA-mediated communication mechanisms among holobiont members would require assessing and expanding knowledge regarding the following topics:

a. Characterization of the role of mobile ncRNAs in the homeostasis of the multi-species consortia by taking advantage of new models and the availability of high-throughput data from different holobionts [120,148].

b. The development of simple holobiont models by a combination of a limited number of species with a supporting host and improvement of the already existing ones to determine a clear picture of the roles of mobile ncRNAs under physiological and homeostatic conditions rather than considering the pathological phenotypes or imbalanced consortia [149,150,151].

c. Inclusion of studies about specific subgroups of neglected organisms belonging to the microbiota, including viruses (virome) and fungi (mycobiome), whose importance is starting to be recognized [152].

d. Characterization of dynamic relationships between different species in model holobionts by the application of the Systems Biology point of view, with the help of artificial intelligence algorithms for the integration of “omics” data, together with environmental factors [101,153].
